# ICAM-1-carrying targeted nano contrast agent for evaluating inflammatory injury in rabbits with atherosclerosis

**DOI:** 10.1038/s41598-021-96042-y

**Published:** 2021-08-13

**Authors:** Ping Li, Lin Jin, Lan Feng, Yingchun Wang, Rong Yang

**Affiliations:** 1 Department of Ultrasound, Jiading District Central Hospital Affiliated Shanghai University of Medicine and Health Sciences, Shanghai, 201800 China; 2 Department of Pathology, Jiading District Central Hospital Affiliated Shanghai University of Medicine and Health Sciences, Shanghai, 201800 China

**Keywords:** Diseases, Molecular medicine

## Abstract

To investigate the feasibility of using ICAM-1-targeted nano ultrasonic contrast to evaluate the degree of inflammatory injury at different stages in the abdominal aorta of rabbits with atherosclerosis (AS). Twenty-five experimental rabbits were assigned to five groups: the control group (A); the week-4 after modeling group (B); the week-8 after modeling group (C); the week-12 after modeling group (D); the week-16 after modeling group (E). All groups were given 2D ultrasonography, conventional ultrasonic contrast (SonoVue), and ICAM-1-targeted nano ultrasonic contrast, respectively. Signal intensity of vascular perfusion was evaluated. Signal intensity of ICAM-1-targeted nano ultrasonic contrast was substantially enhanced and prolonged in the vascular wall of the abdominal bubble aorta increased in B, C, D, and E groups (all *P* < 0.05). A positive linear correlation between intensity and the expression of ICAM-1 (r = 0.895, *P* < 0.001). The intensity of outer membrane was enhanced from week 4 to week 12, and both the intima-media membrane and outer membrane were enhanced with double-layer parallel echo at week 16, which was in line with the progression of atherosclerotic plaque inflammatory injury. ICAM-1-targeted nano contrast agent would be possibly a novel non-invasive molecular imaging method for plaque inflammatory injury and site high expression of specific adhesion molecules in early atherosclerotic lesions.

## Introduction

Stroke has become the second leading cause of death from cardiovascular or cerebrovascular diseases worldwide, whereas the rupture of vulnerable plaques has been found to be closely related to the occurrence of acute cardiovascular diseases^1^. During the development of atherosclerosis (AS), the persistence of inflammation-related factors, the degree of vascular nourishment by the outer membrane, and the degree of neovascularization in plaques are closely related to plaque vulnerability^2^. Previous studies have shown that the intercellular adhesion molecule 1 (ICAM-1) can be highly expressed on the surface of damaged vascular endothelial cells and that its upregulation has a key role in the inflammatory response^3^.

The emergence of nanobubbles leads to revolutionary advances in ultrasonic molecular imaging technology and breaks the limitations of conventional ultrasonic contrast agents developing in the blood pool, making target tissue imaging outside the blood pool feasible^4, 5^. The aim of this experimental study was to explore the feasibility and significance of ICAM-1-targeted nano contrast agent in the evaluation of inflammatory injury in atherosclerotic plaques.

## Materials and methods

### Experimental animals

A total of 25 male New Zealand big-eared rabbits, 4 to 5 months old, weighing 2.0 to 2.6 kg, purchased from Shanghai Jiagan Biotechnology Co., Ltd., with animal production license numbered SCXK (H.) 2015-0005, were used in this study. The rabbits were fed in cages with automatic washing that were automatically washed once every 4 h. The temperature and humidity of the feeding room were recorded, and the room ventilation was checked every morning and afternoon.

### AS rabbit models

The rabbits were grouped into five following groups, and 5 rabbits in each group: group A, the preoperative control group; group B, the week-4 after modeling group; group C, the week-8 after modeling group; group D, the week-12 after modeling group; and group E, the week-16 after modeling group. AS rabbit models were built via high-fat cholesterol feed and surgical balloon injury^6^. Every day, all experimental rabbits were given 120 g of high-fat cholesterol feed that contained 75.5% of basal rabbit feed, 10% of lard, 10% of yolk powder, 4% of cholesterol (of 95% purity), and 0.5% of cholate. After one week of adaptive feeding, the rabbits were subjected to balloon injury in the abdominal aorta. Before the surgery, the rabbits were anesthetized by intramuscular injection of 0.25/kg of suxinmian II (cetirizine hydrochloride injection, Jilin Shengda Animal Medicine Co., Ltd.); the hair from the left lower limb was removed, and the femoral artery was separated. During the surgery, a portable ultrasonic machine (M-Turbo, SonoSite, California) was used to guide the balloon catheter to 2 cm above the level of the abdominal aortic, renal artery (of a feeding length of about 20 cm). After that, the balloon was injected 7.5 atm of air and dragged down to the bifurcation of the abdominal aorta and common iliac artery, which was repeated four times.

### Preparation of ICAM-1-targeted nanobubbles

Dipalmitoyl phosphatidylcholine (DPPC) and biotin-dipalmitoyl phosphatidylethanolamine (DSPE-PEG (2000) Biotin) were mixed in a certain proportion, and 1 ml of hydration solution was added and fixed in a 37 °C constant temperature shaker. Substances were fully shaken, cooled for 20 min in a 4 °C refrigerator. After stratification, 5 ml Perfluoropropane (C_3_F_8_) gas was slowly injected, and vibration for 40 s, then, a milky white suspension was formed. The upper layer of milky white turbidity and lower layer of clear liquid were removed. The middle layer of milky white liquid was added with 1 ml PBS buffer. Then, nano lipid microbubbles were prepared. ICAM-1-targeted nanobubbles were prepared by the avidin–biotin binding method. Streptavidin (10 mg/ml) was added into lipid microbubbles for 30 min, slight oscillation and PBS buffer washing. Then 40 ul of Biotin/FITC-ICAM-1 antibodies were added into the nano lipid microbubbles (800 ul/vial), and the vial was fully suspended for 10 s in a scroll oscillator and placed at room temperature for 30 min for incubation. After that, it was placed in a centrifuge to remove unbound antibodies.

### Measurement of ICAM-1-targeted nanobubbles characteristics

Particle size and concentration of MBs were measured with particle size analyzer (Beckman Coulter, USA), and the average surface potential was measured with Zeta potentiometer (Malvern, England). The experiments were repeated three times. A fluorescence microscope (Olympus, Japan, 400x) was used to observe the binding of nanobubbles to ICAM-1, and a flow cytometer (Becton, Dickinson and Company, USA) was used to measure the binding rate of Biotin/FITC-ICAM-1 to nanobubbles.

### 2D ultrasonography

An ultrasonic diagnostic unit (Toshiba, Aplio400, Japan) was used at a probe transmission frequency of 14 MHz, depth of 2 cm, and frame rate of 32 frames/s. 2D ultrasonography was used to measure the thickness of the intima-media membrane at the site in the abdominal aorta that was the thickest. Color Doppler ultrasonography was used to observe blood filling in the lumen.

### SonoVue ultrasonic contrast

The rabbits were anesthetized by intramuscular injection of 0.1 ml/kg of suxinmian II (cetirizine hydrochloride injection, Jilin Shengda Animal Medicine Co., Ltd.), placed at the supine position on the operating bench; the hair was removed from the abdominal wall, and an indwelling needle (20G, Intima IITM, Suzhou Becton Dickinson Medical Devices Co., Ltd.) was placed in the left auricular vein. SonoVue (Bracco, Italy) was used as the ultrasonic contrast agent. An ultrasonic diagnostic unit (Toshiba, Aplio400, Japan) was used at a probe transmission frequency of 14 MHz, depth of 2 cm, a frame rate of 10 frames/s, and mechanical index of 0.08. Before use, 5 ml of 0.9% NaCl (China Otsuka Pharmaceutical Co., Ltd.) was injected into the bottle and shaken into microbubble suspension. After that, 0.1 ml/kg of the contrast agent was extracted and quickly injected through the auricular vein, which was followed by an injection of 1 ml of 0.9% NaCl.

### ICAM-1-targeted nano ultrasonic contrast

All experimental rabbits were given ICAM-1-targeted nano ultrasonography under the same ultrasonic conditions as described above 1 h after SonoVue ultrasonic contrast after being anesthetized in the same way as described above. Bolus injection of 0.7 × 10^8^ MBs/kg ICAM-1-targeted nanobubbles was quickly given to the rabbits at the site of the indwelling needle through the auricular vein. After that, the rabbits were immediately injected with 1 ml of 0.9% NaCl to rinse the catheter, and continuous dynamic images were acquired in the contrast mode. Real-time imaging was performed at 2 s after intravenous injection of ICAM-1-targeted nanobubbles and images were analyzed in the built-in software program.

### Serum lipid test

After ultrasonic examinations, 5 ml blood was drawn from the ear vein of experimental rabbits. The whole blood was centrifuged at 4 °C for 15 min for serum. Enzymes were used to measure the total cholesterol (TC), triglyceride (TG), high-density lipoprotein (HDL), and low-density (LDL) in the serum samples.

### Histopathology and Immunohistochemistry

Hematoxylin and eosin (HE) staining: The extracted abdominal aorta was sealed in 5% formalin solution and embedded in paraffin, which was then cross-sectioned for a transverse section of tissues of about 3 μm in thickness. The section was then HE stained to observe the degree of atherosclerotic lesions.

Western Blot: The BCA protein concentration quantitative kit (Thermo, USA) was used to measure the expression of ICAM-1 in the abdominal aorta, and the image analysis system (Quantity One, Bio-Rad, California) was used to analyze the optical density of the target.

Immunohistochemistry staining: Specimens were immunostained with an anti-ICAM-1 antibody (1:100 dilution, Bioss Beijin) for the co-localization of ICAM-1-targeted ultrasonic nanobubble and inflammatory injury at atherosclerotic lesions. Immunohistochemical reagents and secondary antibodies were from Maixin Fuzhou.

### Statistical analysis

SPSS 19.0 (IBM, Armonk, NY, USA) was used for data analysis. Continuous variables were expressed as means ± standard deviation (SD). Independent samples t-test was performed for between-group comparisons, and one-way analysis of variance (ANOVA) followed by post-hoc LSD test for multiple-group comparisons. Pearson correlation analysis was used to analyze the correlation. The level of statistical significance was set at *P* < 0.05.

### Ethical approval

This study was carried out in accordance with ARRIVE guidelines, and the study was approved by the Animal Experimental Ethical Inspection of Shanghai Jiagan Biotechnology Co., Ltd. (No.: JGLL20181208). All animal experiments were performed in accordance with the Guide for the Care and Use of Laboratory Animals.

## Results

All rabbits were successfully given ultrasonic contrast and sampled for pathological tests.

### Serum lipid

The level of TC, TG, LDL, and HDL in groups B,C,D and E were higher than the control group, there were significant statistically differences among the groups (P < 0.05). The results are shown in Table 1.

**Table 1:** Comparison of serum lipid in experiment rabbits (mmol/L).

	TC	TG	LDL	HDL
Control group	1.67 ± 0.14	1.11 ± 0.09	1.27 ± 0.25	0.59 ± 0.09
Week-4 group	12.84 ± 3.38*	1.85 ± 0.25*	9.14 ± 2.92*	0.75 ± 0.09*
Week-8 group	22.73 ± 3.97*^#^	3.14 ± 0.57*^#^	15.03 ± 3.86*^#^	1.24 ± 0.30*^#^
Week-12 group	32.49 ± 4.86*^#△^	4.71 ± 1.12*^#△^	19.13 ± 1.21*^#△^	1.66 ± 0.14*^#△^
Week-16 group	38.54 ± 4.30*^#△□^	8.49 ± 1.43*^#△□^	22.46 ± 1.88*^#△□^	1.96 ± 0.17*^#△□^
*F* value	79.464	58.237	61.963	54.422
*P*	< 0.001	< 0.001	< 0.001	< 0.001

*P < 0.05 versus control group; ^#^*P* < 0.05 versus week-4 group; ^△^*P* < 0.05 versus week-8 group; ^□^*P* < 0.05 versus week-12 group.

### Binding of ICAM-1 antibodies to ultrasonic nanobubbles

**Figure 1:**
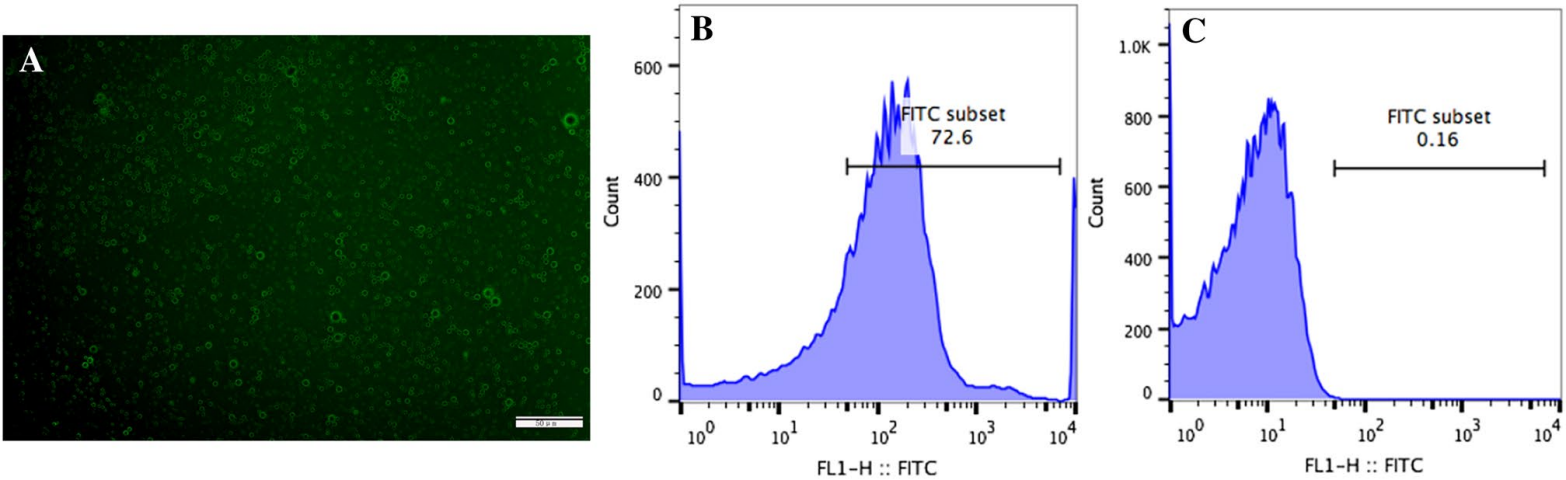
Images of ICAM-1-targeted ultrasonic nanobubbles under the fluorescence microscope (**A**). Scale bar shows 50 μm. Flow cytometry of microbubbles binding with ICAM-1 antibodies (**B**) and without ICAM-1 antibodies (**C**).

Particle analyzer showed that the ICAM-1-targeted nanobubbles used in this study has a median diameter of (683.33 ± 25.38) nm, the range was between 655 and 704 nm and the concentration was about (2.39 ± 0.15) × 10^8^/ml. Zeta potentiometer revealed that the average surface potential was –(5.58 ± 1.29) mV. A fluorescence microscope showed that nanobubbles bound to ICAM-1 antibodies showed green fluorescence rings on the surface (Fig. 1A). Flow cytometry revealed that about 72.6% of the microbubbles were successfully conjugated with ICAM-1 antibody (Fig. 1B). Flow cytometry of naked bubble was showed in Fig. 1C.

### 2D ultrasonography imaging findings

**Figure 2:**
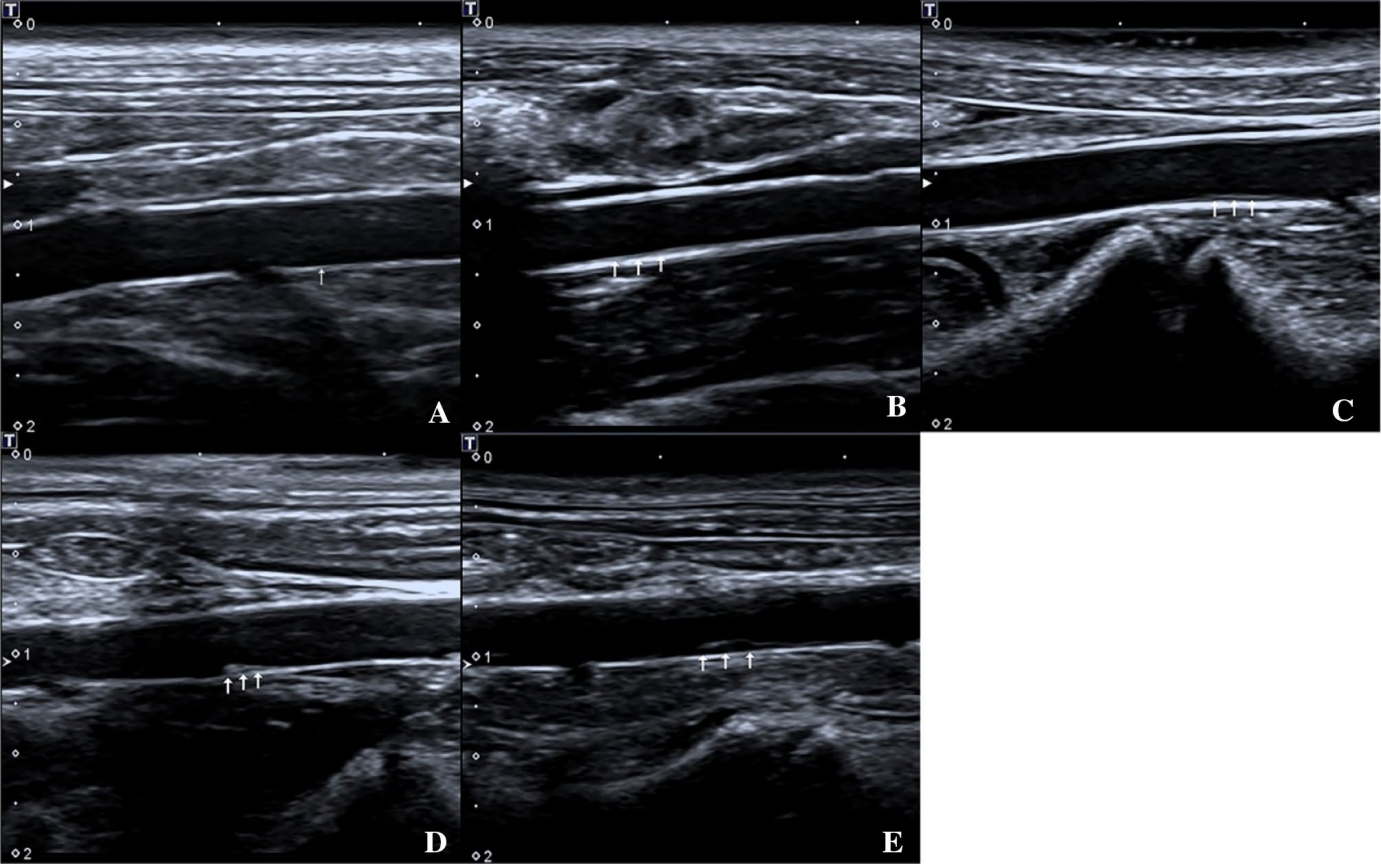
Two-dimensional ultrasonography images of the abdominal aorta of rabbits in different groups, five rabbits in each group. (**A**) Control group; (**B**) Week-4 group; (**C**) Week-8 group; (**D**) Week-12 group; (**E**) Week-16 group. The intima-media membrane and the plaque on the wall was indicated by the arrows.

The abdominal aortic was observed by ultrasonography in all groups, and images are shown in Fig. 2. A well-filled blood flow in the abdominal aortic lumen and a thin and smooth intima-media membrane were observed in group A. B group showed enhanced intima-media echo in the vessel and no obvious atherosclerotic plaque formation. C group had a slightly thickened intima-media membrane and no obvious atherosclerotic plaque formation. D group and E group showed gradual thickening of intima-media membrane in the abdominal aorta and different atherosclerotic plaque formation degrees. The intima-media membrane thickness (IMT) displayed a significant increase among the groups (*P* < 0.05) (Group A: 0.08 ± 0.02 mm; group B: 0.17 ± 0.02 mm; group C: 0.23 ± 0.02 mm; group D: 0.30 ± 0.03 mm; group E: 0.43 ± 0.05 mm). The results are shown in Fig. 5A.

### SonoVue ultrasonic contrast imaging findings

**Figure 3:**
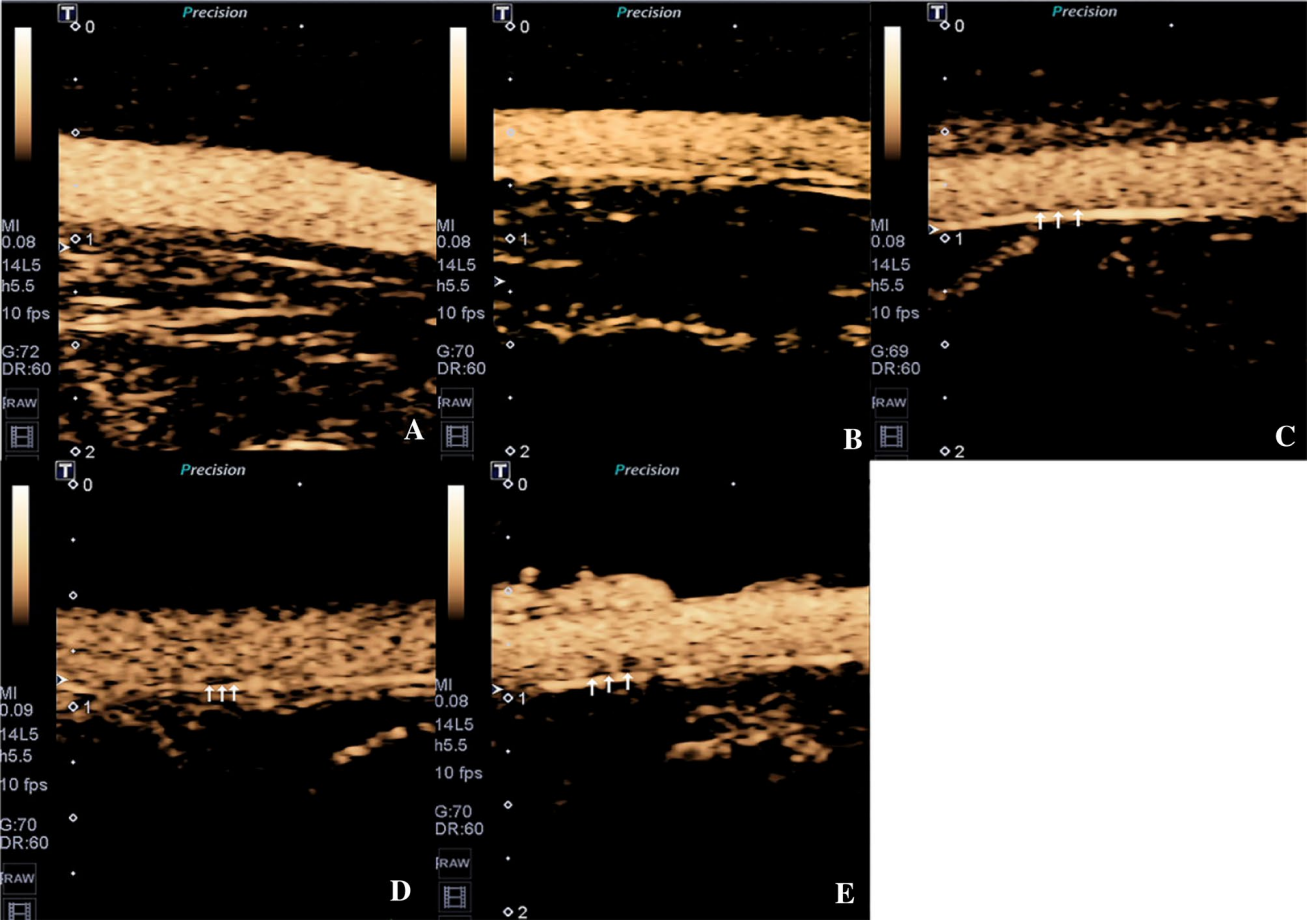
SonoVue ultrasonic contrast images of abdominal aorta of rabbits in different groups, five rabbits in each group. (**A**) Control group; (**B**) Week-4 group; (**C**) Week-8 group; (**D**) Week-12 group; (**E**) Week-16 group. The plaque on the wall was indicated by the arrows.

Figure 3 shows a representative abdominal aortic ultrasound images from rabbits with varying degrees of atherosclerosis after the injection of SonoVue. Group A had well-filled contrast agents in the abdominal aortic lumen and smooth vascular wall; group B had no obvious abnormalities compared with the control group; group C had well-filled contrast agents in the lumen and slightly thickened intima-media membrane; group D and E had well-filled contrast agents in the lumen and different degrees of filling defects. The maximum thickness of the intima-media membrane of groups B,C,D,E increased gradually compared to the group A (*P* < 0.05) (Group A: 0.09 ± 0.01 mm; group B:0.18 ± 0.03 mm;group C: 0.23 ± 0.02 mm;group D: 0.32 ± 0.03 mm; group E:0.44 ± 0.41 mm). The results are shown in Fig. 5B.

### ICAM-1-targeted nano ultrasonic contrast imaging findings

**Figure 4:**
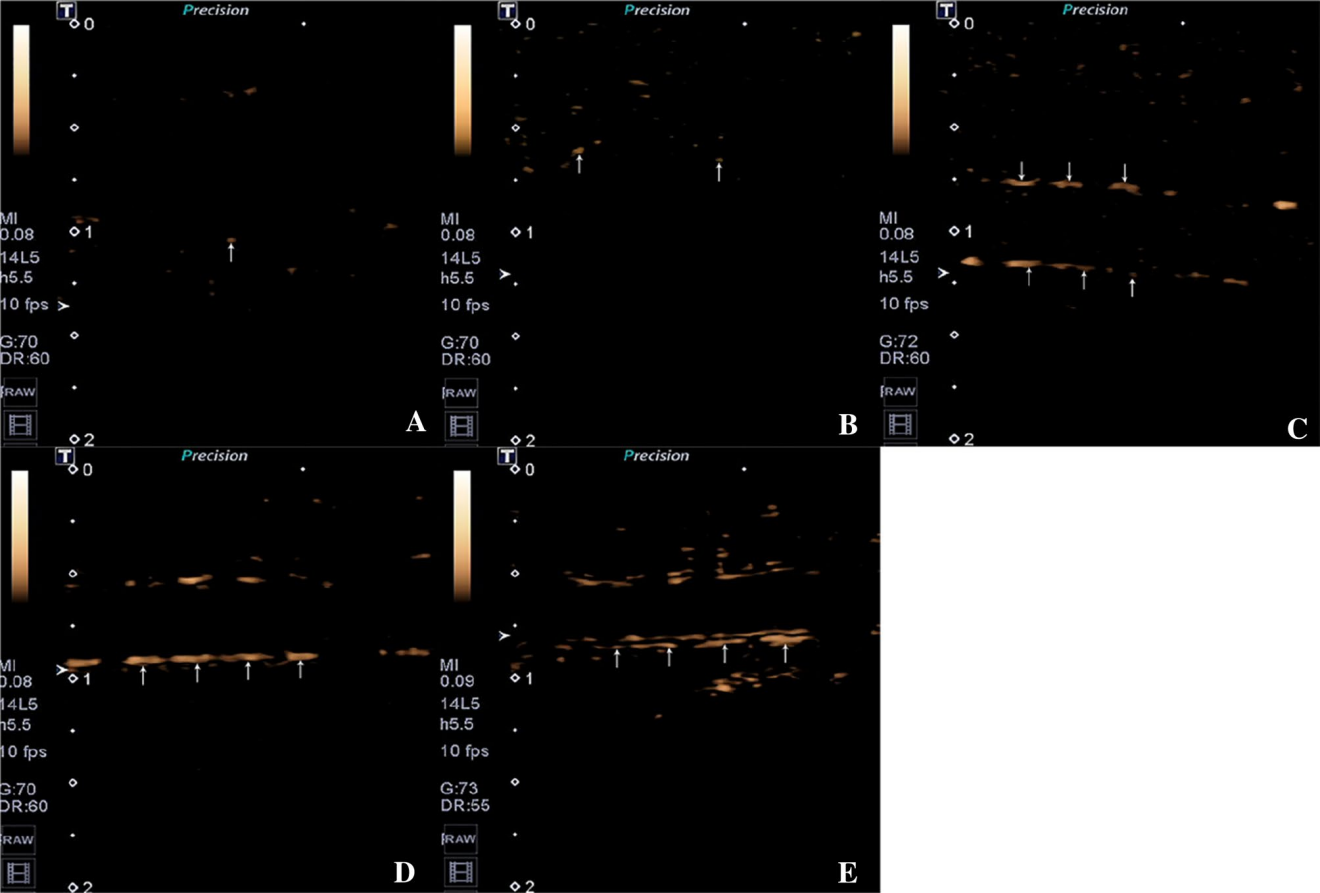
ICAM-1-targeted nano ultrasonic contrast images of abdominal aorta of experiment rabbits in different groups, five rabbits in each group. (**A**) Control group; (**B**) Week-4 group; (**C**) Week-8 group; (**D**) Week-12 group; (**E**) Week-16 group. The vascular wall was indicated by the arrows.

**Figure 5:**
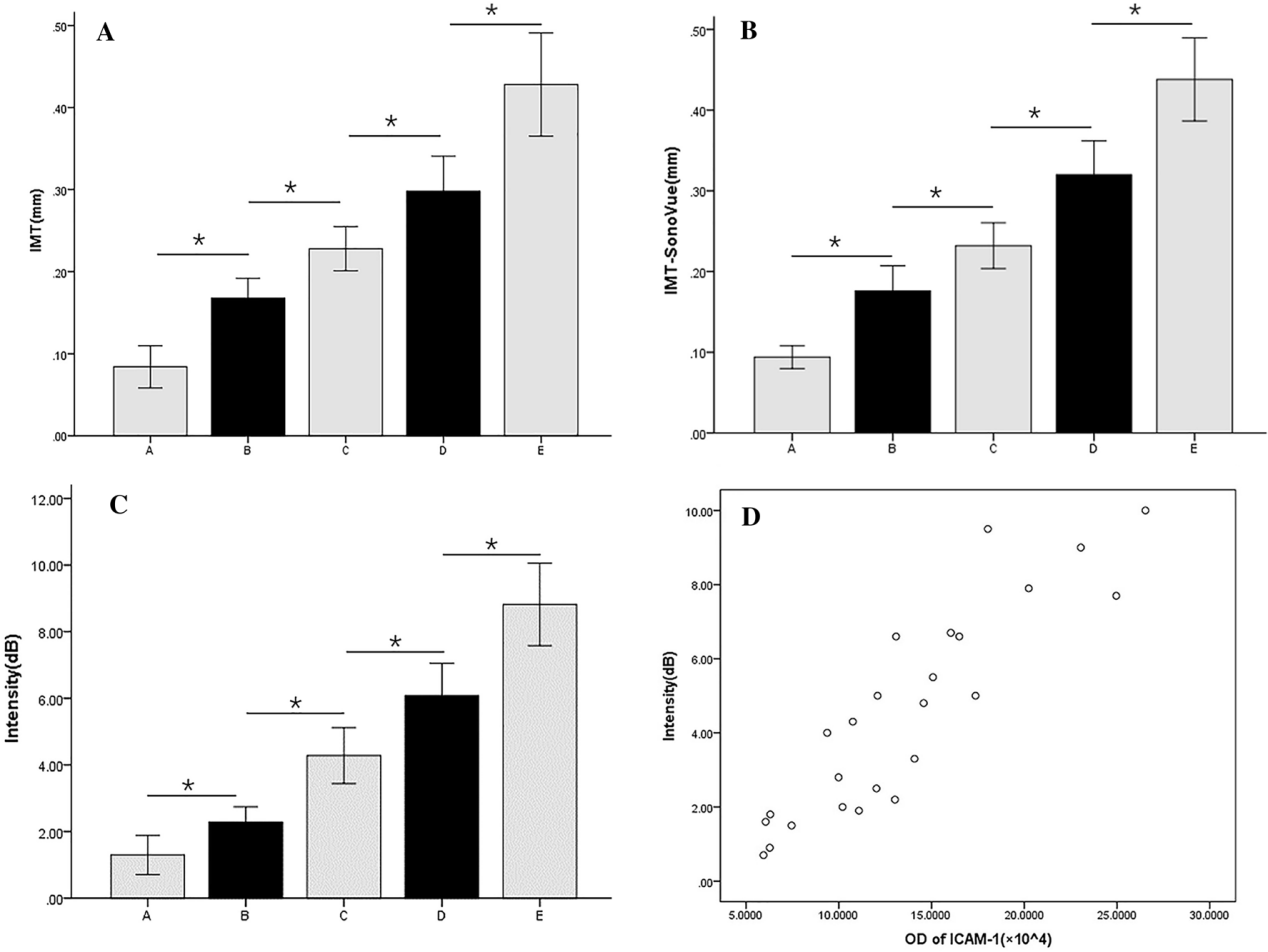
Comparison of the intima-media membrane thickness of 2D (**A**), SonoVue ultrasonic contrast (**B**) and intensity of abdominal wall of experiment rabbits of ICAM-1-targeted nano ultrasonic contrast (**C**), five rabbits in each group. *Indicates significant difference from the groups *P < 0.05, all values presented as mean ± standard deviation. The correlation analysis of the OD of ICAM-1 and the vascular wall intensity (D). Data analysis and images were created by using SPSS 19.0 (IBM, Armonk, NY, USA).

**Figure 6:**
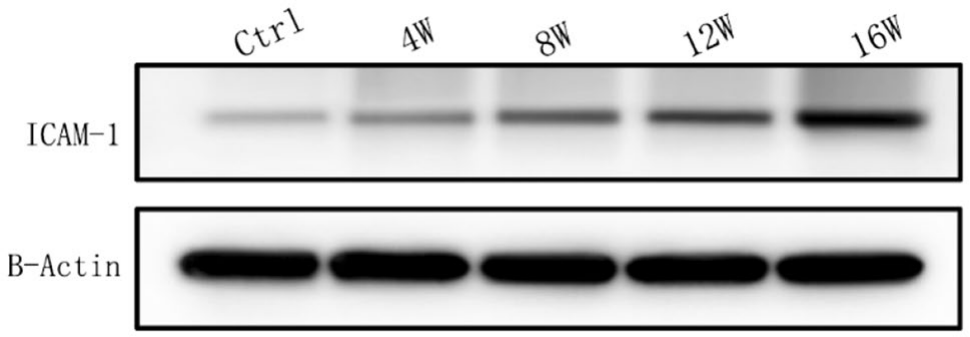
Western blots show expression of ICAM-1 of rabbit abdominal aorta. Full-length blots are presented in Supplementary Figure S1.

After injection, the microbubbles in the aortic lumen circulation was rarely seen in all groups. The vascular wall of the group A and group B were not obviously enhanced in the whole process. Group C, D, and E groups had the contrast agent completely faded in the vascular lumen 2 min after the injection of contrast agent, as well as continuously enhanced imaging of the vascular wall. As the modeling time extended, the imaging intensity of the vascular wall increased, and the fading time was delayed. Briefly, E group showed that after 2 min, targeted nanobubbles signal intensity retained in the intima-media membrane and the outer membrane was significantly higher when compared with the other groups and presenting double-layer parallel echo (Fig. 4). There were statistically significant differences in the vascular wall intensity (*P* < 0.05) (Group A: 1.30 ± 0.47 dB; group B: 2.28 ± 0.37 dB; group C: 4.28 ± 0.68 dB; group D: 6.08 ± 0.78 dB; group E: 8.82 ± 1.00 dB). The results are shown in Fig. 5C. The above data suggested that a specific retention of ICAM-1-targeted nanobubbles in the intima-media membrane and the outer membrane. In other words, the signal enhancement and its dynamic change with time of ICAM-1-targeted nanobubbles could be considered as novel parameters in identifying inflammatory injury. Consistent with the western blot analysis, it further confirmed the increase of ICAM-1 level in the aortic (Fig. 6). Correlation analysis showed that the OD value of ICAM-1 was closely correlated with intensity of vascular wall with targeted ultrasonic contrast (r = 0.895, *P* < 0.001). The results are shown in Fig. 5D.

### Histopathology and immunohistochemistry findings

**Figure 7:**
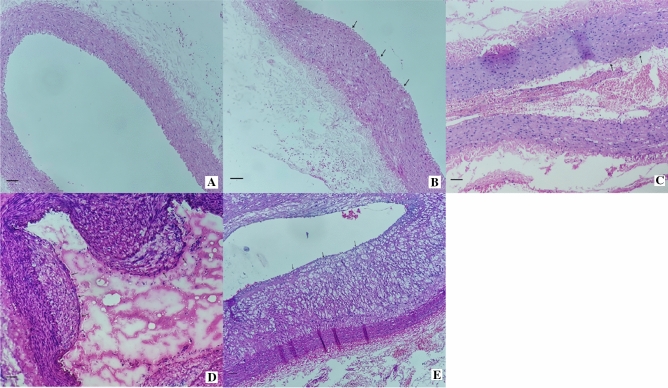
Rabbit abdominal aorta stained by hematoxylin and eosin (× 100). (**A**) Control group; (**B**) Week-4 group; (**C**) Week-8 group; (**D**) Week-12 group; (**E**) Week-16 group. scale bar shows 100 μm.

After HE staining, the control group had a complete arrangement of the vascular endothelium and orderly and long-fusiform arrangement of smooth muscle cells (SMCs) in the abdominal aorta and no vascular lipid deposition; group B had thickened smooth muscle layer of the medial membrane in the vascular wall, and disorderly arrangement of SMCs; C and D groups had obviously proliferated SMCs in the medial membrane, proliferated foam cells and disorderly arrangement of cells, and E group had a large number of foam cells accumulated into atherosclerotic plaques below the endothelial cells (Fig. 7).

**Figure 8:**
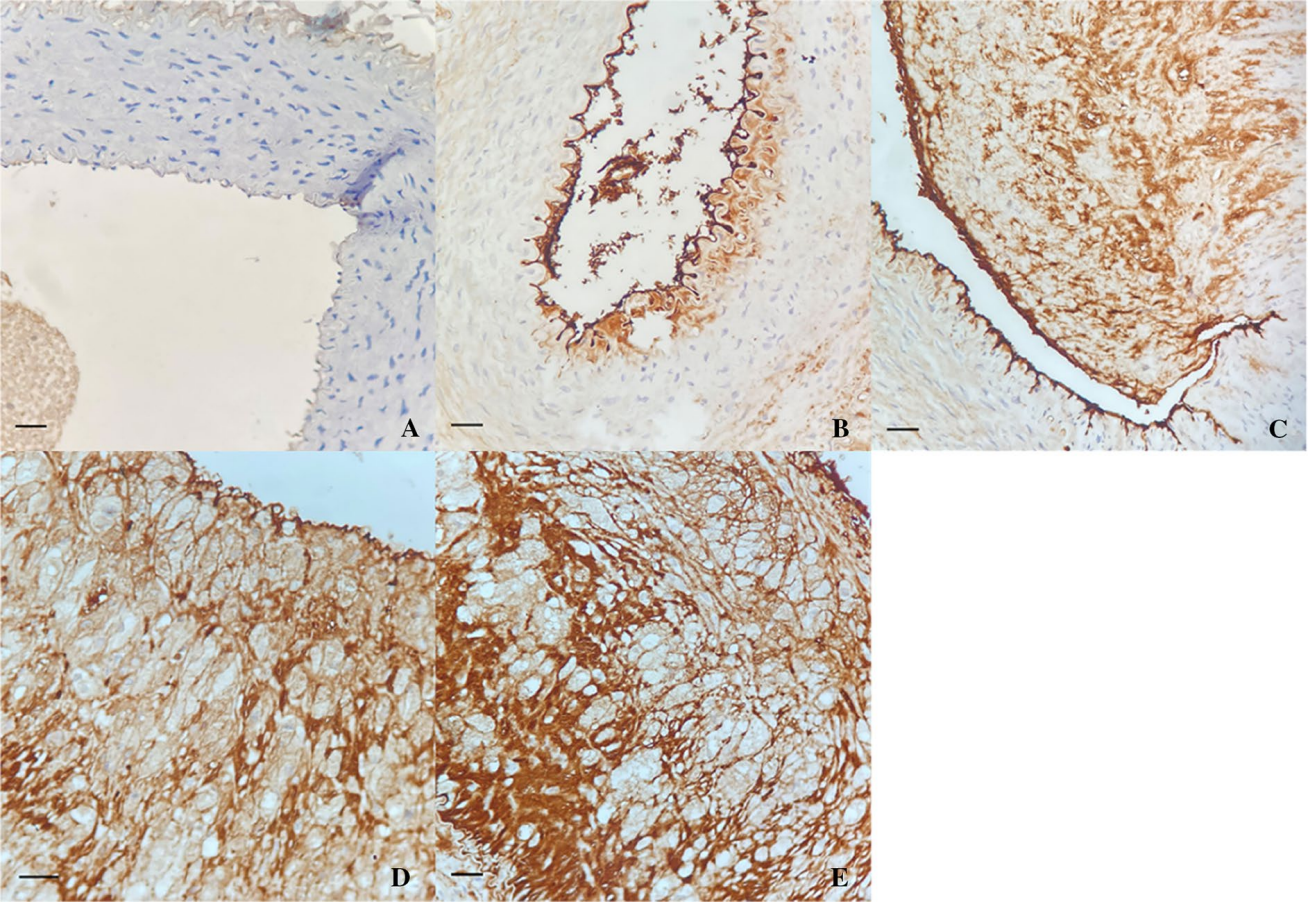
Expression of ICAM-1(× 400). Yellowish-brown granules were deposited in vascular endothelialcells, smooth muscle cells, macrophages and foam cells. Control group stained negative for ICAM-1; Week-4, 8, 12, 16 groups stained positive for ICAM-1. scale bar shows 25 μm.

Immunohistochemistry staining: There was stained negative for ICAM-1 in vascular endothelium in the control group. Then positive staining were gradually expressed in vascular endothelial cells, smooth muscle cells, macrophages and foam cells in group B,C,D,E. The percentage of positive cells gradually increased, and the staining intensity gradually deepened (Fig. 8).

## Discussion

AS, one of the common diseases in clinical practice, is an inflammatory disease that runs through the disease's whole course, making plaques unstable and prone to rupture or erosion^7, 8^. An essential feature in the early formation of plaques is the recruitment of white blood cells to the vascular wall. In general cases, endothelial cells increase the expression of leukocyte adhesion molecules, such as the expression of intercellular adhesion molecule-1 (ICAM-1), to resist the adhesion of white blood cells in the blood^9^. ICAM-1 is present in 46% of atherosclerotic lesions^10^.Therefore, ICAM-1 is one of the critical inflammatory markers related to AS. Previous studies have shown that up-regulated expression of ICAM-1 is involved in the occurrence and development of AS lesions and that to some extent reflects the degree of inflammatory endothelial injury. Hence, ICAM-1 is a good target for the detection of existing AS lesions, and can serve as a molecular probe reflecting the early imaging diagnosis and treatment of inflammation^11, 12^.

Nanoultrasonic contrast agent, for its small grain size, can freely pass through pathologically new vessels enabling the imaging of new vessels. It can also pass through the endothelial gap of new vessels, enabling extravascular tissue imaging with high sensitivity^5, 13^. After binding to specific antibodies or ligands, ultrasonic contrast agents become targeted contrast agents that can actively bind to targeted areas and highly aggregated in such areas to enable specific imaging^14, 15^. This experimental study was carried out in AS animal models. ICAM-1-targeted ultrasonic nanobubbles were prepared for dynamic observation of the capability of ICAM-1-targeted ultrasonic nanobubbles to recognize atherosclerotic inflammatory lesions at different stages. Our results revealed that ICAM-1-targeted nano contrast agents had a longer time of imaging in the vascular intima-media membrane compared to the lumen, this was specific to the severity of inflammation in atherosclerosis, while conventional micron ultrasonic contrast agents in the lumen faded almost at the same time as lumen wall contrast agents, which was consistent with the study results of Fan et al.^16^. The imaging intensity of ICAM-1-targeted ultrasonic nanobubbles in the vascular wall significantly increased and the imaging time significantly extended with the progression of inflammation. The imaging intensity of the vascular wall in this experimental study revealed a positive linear correlation with the expression of ICAM-1 in tissues. It is possible that targeted nanobubbles were sucked into cells after adhering to activated neutrophils and monocytes but maintained the same acoustic properties. Also, after free microbubbles in the blood circulation were emptied, contrast agents in the lumen faded while intracellular targeted nanobubbles in the vascular intima-media membrane continued to enable ultrasonic imaging. Another possible reason might lie in the correlation between microbubble imaging's time and intensity with the site and severity of inflammation.

A previous study showed that new vessels in plaques gradually grew from the outer membrane to the atherosclerotic lesion site, providing a new pathway for monocytes and immune cells. Meanwhile, SMCs in the artery migrated into the medial membrane and proliferated, gradually forming fibrous caps. Also, the rupture was closely related to plaque vulnerability^17, 18^. As shown in this experimental study, ICAM-1-targeted ultrasonic nanobubbles had delayed imaging in the outer membrane of the vascular wall at week 4 after modeling, and enhanced imaging, with double-layer parallel echo, in both the intima-media membrane and outer membrane of the vascular wall at week 16 after modeling. Consequently, we speculated that ICAM-1-targeted nanobubbles could specifically bind to ICAM-1 targets on the abdominal aortic wall. Also, their site of adherence in plaques was the same as the site vulnerable to atherosclerosis.

The limitations of this study need to be mentioned. First, the sample size used in this study is small, and it needs to be increased in the future. Second, the binding rate of ICAM-1 antibody to nanobubbles is not high, and the preparation conditions should be further optimized. In addition, our image acquisition is based on 2D long-axis section of vessels and it should be further combined with the short axis section in the future to make the observation more comprehensive.

## Conclusion

In this study, ICAM-1-targeted nanobubbles were fabricated and used as agents for ultrasonic molecular imaging. ICAM-1-targeted ultrasonic nanobubble contrast agent could reflect the degree of inflammatory injury at different stages of atherosclerotic lesions, verified by pathologic results. The method might be potentially applied to reveal inflammatory changes of target lesions at the molecular level. Capable of showing molecular changes of inflammation, this novel imaging method may be promising in the early diagnosis and accurate evaluation of degree of inflammatory injury in atherosclerotic lesions.

## Supplementary Information

Supplementary Figure S1.
