# Supplementary figures and images for: ICAM-1-carrying targeted nano contrast agent for evaluating inflammatory injury in rabbits with atherosclerosis
Source: Sci Rep. 2021 Aug 13;11:16508. doi: 10.1038/s41598-021-96042-y (PMC8363608; doi:10.1038/s41598-021-96042-y)

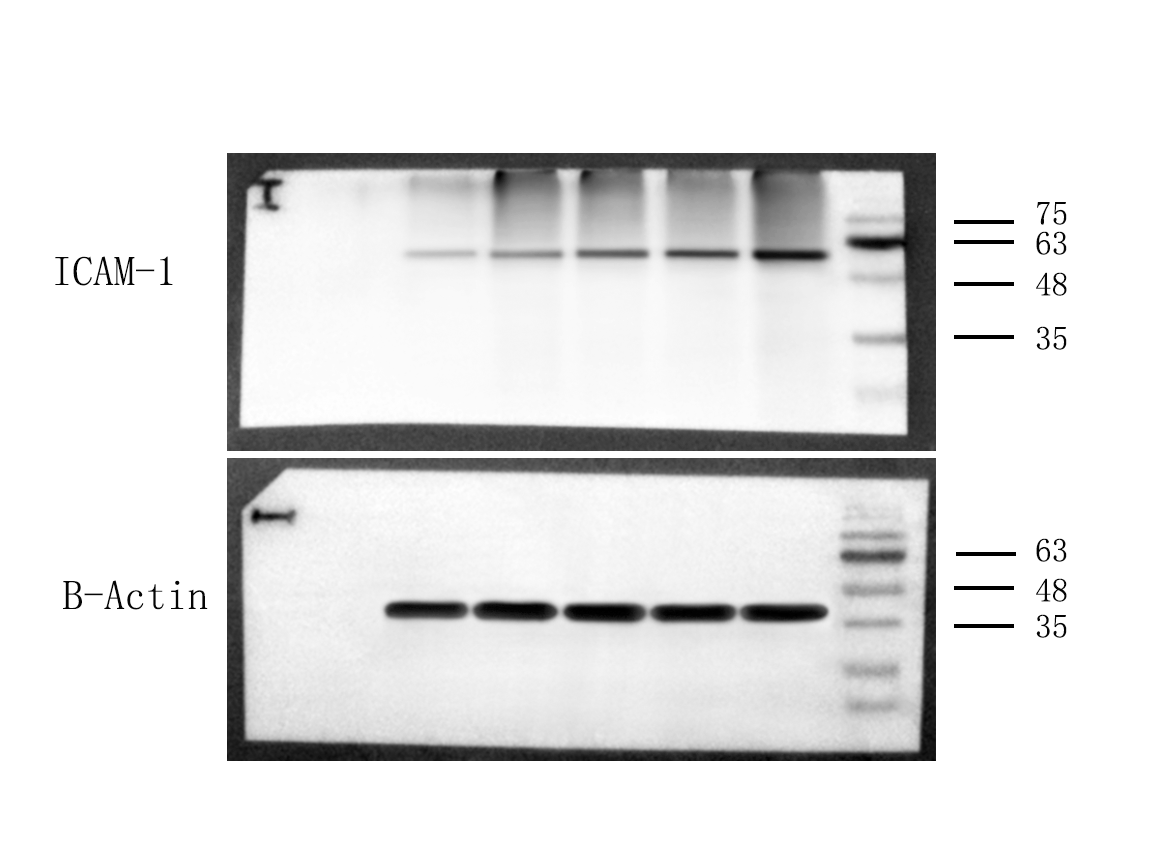

Supplement: Supplementary file 1 — Supplementary Figure S1. [file 41598_2021_96042_MOESM1_ESM.tif]
